# Heterotopic cervical pregnancy after *in-vitro* fertilization - case report and literature review

**DOI:** 10.5935/1518-0557.20190017

**Published:** 2019

**Authors:** Maria Eduarda Furtado Fernandes Terra, Luiz Augusto Giordano, Mário Vicente Giordano, Renato Augusto Moreira de Sá, Fernanda Campos, Isaac Moise Yadid, Felipe de Oliveira Pinto

**Affiliations:** 1 Universidade Federal do Estado do Rio de Janeiro (UNIRIO) – Rio de Janeiro – RJ - Brazil; 2 Universidade Federal Fluminense (UFF) – Niteroi – RJ - Brazil; 3 Clínica Primordia Medicina Reprodutiva – Rio de Janeiro – RJ - Brazil

**Keywords:** cervix uteri, *in vitro* fertilization, pregnancy, heterotopic

## Abstract

Heterotopic cervical pregnancy is an uncommon condition, with a rising incidence due to the increasing number of pregnancies resulting from *in-vitro* fertilization (IVF). Although it is associated with maternal-fetal complications, there is no consensus in the literature about the best approach for this condition. This study aims to report a case of cervical heterotopic gestation after IVF in which the intrauterine pregnancy was preserved, with spontaneous elimination of the cervical gestational sac after patient sedation and introduction of the vaginal speculum. In addition, we reviewed the literature on the subject, which demonstrated that most cases have a favorable outcome, especially after treatment with surgical excision of the cervical pregnancy. The growing body of evidence is still scarce to define the best treatment for this condition.

## INTRODUCTION

Heterotopic pregnancy refers to the presence of simultaneous pregnancies at two different implantation sites, which commonly consists of an association between intrauterine (IU) and ectopic pregnancies. The uterine cervix represents the most rare site of implantation of an heterotopic pregnancy, with an estimated incidence of 1:30,000 pregnancies (Reece *et al.*, 1983). However, this number is rising, which is thought to be associated with the growing popularity of assisted reproduction technology, reaching a frequency of 1% of the pregnancies resulting from these methods (Dibble & Lourenco, 2016).

Heterotopic cervical pregnancy is a rare event and its occurrence is related to a higher incidence of maternal-fetal complications, such as prematurity, severe bleeding and emergency hysterectomy (Molinaro & Barnhart, 2018; Kim *et al*., 2012; Tsakos *et al*., 2015). Considering that most of the affected patients were submitted to *in vitro* fertilization (IVF) due to infertility, treating cervical pregnancy maintaining the IU embryo and the patient’s fertility becomes a priority and a challenge to the practice of obstetrics.

The current literature on heterotopic cervical pregnancy is still scarce and there is no consensus about the best treatment for this condition. Therefore, this study aims to report a case of a post-IVF heterotopic cervical pregnancy whose treatment enabled the preservation of IU pregnancy. In addition, we present a review of the recent literature on the subject in order to help establish standards of treatment for this increasingly frequent condition in medical practice. About to our knowledge this is the first case described in Brazil.

## CASE DESCRIPTION

A 39-year-old woman from Rio de Janeiro - Brazil sought specialized medical care early in 2015 due to secondary infertility. She reported a history of 5 previous spontaneous abortions with gestational age of around 5/6 weeks, including a tubal pregnancy (right tube), which was treated with methotrexate (MTX). In addition, she had once undergone a hysteroscopic myomectomy. The patient presented a transvaginal ultrasound (US) without significant alterations and a hysterosalpingography showing bilateral tube patency and mucosal thickening of the right tube infundibulum.

The initial diagnostic evaluation consisted of spermogram, sperm DNA fragmentation, hormonal dosages (LH, FSH, estradiol, prolactin, TSH, T4, insulin), glycemia, serologic tests (toxoplasmosis, rubella, cytomegalovirus, HIV, syphilis and viral hepatitis B and C), antithyroid antibodies (anti-TPO and anti-thyroglobulin), screening for thrombophilia, hysteroscopy and karyotype of the couple. None of the exams revealed clinically significant changes.

Between July 2015 and July 2016, the patient was submitted to three IVF procedures after ovulation induction with gonadotropins and none of them resulted in pregnancy. Therefore, oocyte donation therapy was proposed. In March 2017, two donated frozen embryos were transferred with no success. In October 2017, she was submitted to a new IVF cycle with oocytes from another donor and six embryos were obtained. The transfer of the first two embryos did not result in pregnancy. At this point, an Endometrial Receptivity Array (ERA) was performed, which showed a receptive endometrium. Then, two more embryos were transferred with no pregnancy outcome. Thereafter, it was decided to empirically use immunoglobulin during the transfer of the last two embryos and we finally had a pregnancy.

**Figure 1:**
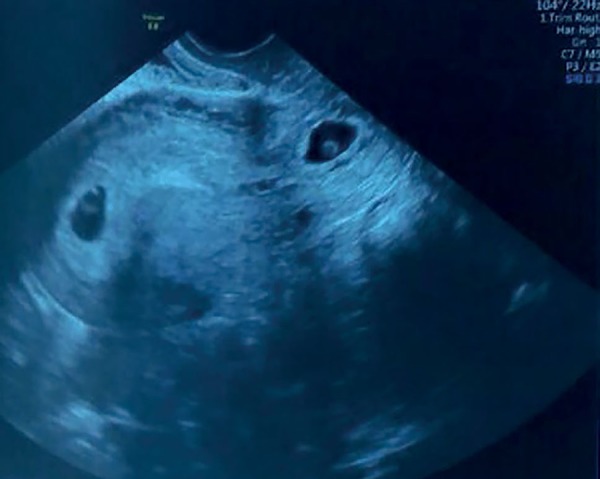
Transvaginal US (10 weeks of gestational age)

The first trimester transvaginal US showed an embryo compatible with 7 weeks and 5 days of gestational age, normoimplanted, with a heartbeat, associated to a hypoechoic endocervical image. Since the patient was asymptomatic, we opted for expectant management, with a new US in 2 weeks. The following US (Figure 1) showed a 10-week intrauterine embryo and an endocervical hypervascularized echogenic image of 19 x 17 mm, compatible with a cervical gestation.

**Figure 2:**
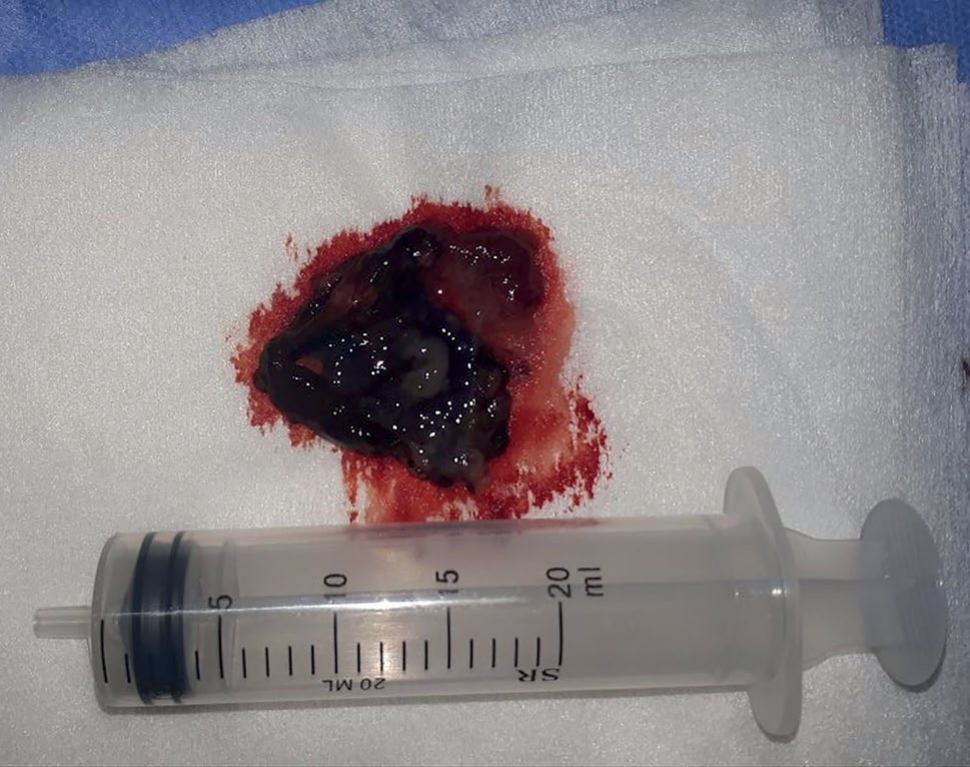
Trophoblastic tissue from cervical os

The proposed treatment would be the selective reduction of ectopic gestation with US guided Chloride Potassium (KCl) injection in the cervical gestational sac under general anesthesia. However, after anesthetic induction and introduction of the vaginal speculum to perform the procedure, there was spontaneous elimination of the cervical embryo (Figure 2), with preservation of the IU pregnancy. Discrete cervical curettage was performed for the excision of remaining trophoblastic tissue. The patient presented a satisfactory course, not requiring the use of tocolytics. The IU pregnancy proceeded normally until C-section delivery at 39 weeks, yielding a 3215g and 49 cm baby.

## DISCUSSION

Cervical heterotopic gestation is a rare event but has a rising incidence due to the increasing number of pregnancies resulting from IVF. The reason why IVF predisposes to the anomalous implantation of the embryo is not well established, but it is believed that there is an association between risk factors common to patients submitted to this method (cervical abnormalities and previous curettage) and mechanisms inherent to the technique (volume and viscosity of the transfer medium, reflux of the transferred embryo or cervical trauma during the procedure) (Molinaro & Barnhart, 2018; Pinto *et al*., 2016). The patients’ main symptom was vaginal bleeding and diagnostic confirmation was achieved through US. The treatment options aiming to maintain the IU pregnancy can be divided between surgical excision of the cervical embryo or selective reduction of the cervical embryo by intra-amniotic injection of KCl or MTX. Despite the high potential for maternal-fetal morbidity and mortality, there is no pre-established treatment guideline for this condition.

Thus, we performed a review of the recent literature on cervical heterotopic gestation, aiming to analyze its epidemiology, the different forms of treatment and its relation with a favorable outcome. We used the PubMed database and searched for the terms "pregnancy, heterotopic" and "cervix uteri", as well as later active search in the bibliographic references of the selected papers. We found 37 case reports of cervical heterotopic gestation, in the English and Spanish languages, in addition to the present report (Table 1). The reports vary from year 1989 to year 2018 and there was no case report from Brazil.

**Table 1:** Overview of heterotopic cervical pregnancy case reports

Author (year)	Maternal age	Method of conception	Gestational age at diagnosis	Treatment	Pregnancy outcome	Presenting symptom / Complications
Current paper	39	IVF	7w 5d	Spontaneous elimination of the CG after patient sedation, cervical (US guided)	C-section (39w)	Asymptomatic
Saito *et al.,* 2018	39	IVF	5w 2d	CG extraction with forceps (US guided)	C-section (36w)	Vaginal bleeding / Blood transfusion, total placenta accreta, hysterectomy
Punhani *et al.*, 2016	34	IVF	8w	Local injection of KCl, uterine artery embolization,MTX IM, curettage	Intentional interruption	Vaginal bleeding / Blood transfusion
Pinto *et al*., 2016	35	IVF+ICSI	7w 2d	Cervical curettage (US guided)	C-section (39w)	Asymptomatic
Subedi *et al*., 2016	33	IVF	5w	Uterine artery embolization, aspiration, hysteroscopic removal with forceps	Intentional interruption	Vaginal bleeding
Elena *et al.*, 2016	37	IVF	5w	Aspiration and cervical curettage, cerclage	Vaginal delivery (35.4w)	Asymptomatic / Bleeding, pelvic pain
Tsakos* et al.*, 2015	41	IVF	5w 3d	CG aspiration, Foley catheter insertion, cerclage	C-section (38w)	Asymptomatic
Lin *et al*., 2013	32	IVF	8w	Cervical curettage, electro-cauterization	Maintenance of IU pregnancy (did not specify)	Vaginal bleeding, pelvic pain / Triplet gestation: association with tubal gestation
Uysal & Uysal, 2013	31	Spontaneous	6w	Local injection of KCl, CG aspiration, Foley catheter insertion, cerclage	Delivery (38w)	Vaginal bleeding
Moragianni *et al*., 2012	40	IVF	7w 3d	CG extraction with forceps, Foley catheter insertion, cerclage	C-section (39w)	Vaginal bleeding
Kim *et al*., 2012	36	IVF	5w 2d	CG extraction with forceps (US guided)	C-section (40w 5d)	Vaginal bleeding
Deka *et al.*, 2012	38	IVF	11w 1d	Local injection of KCl and MTX	C-section (36w 4d)	Vaginal bleeding
Faschingbauer *et al.,* 2011	25	Spontaneous / Stimulation (clomiphene citrate)	9w	CG aspiration (US guided), cerclage	Vaginal delivery (39w 3d)	Vaginal bleeding
Sijanovic, *et al*., 2011	30	Spontaneous	6-7w	Local injection of MTX	Vaginal delivery (39w)	Vaginal bleeding
Sánchez-Ferrer *et al.*, 2011	33	IVF	6w 5d	Intra-arterial injection of MTX, uterine artery embolization	Intentional interruption	Vaginal bleeding
Hafner *et al.*, 2010	34	IVF	6w	Foley catheter insertion (US guided), cerclage, ligation of descending cervical branches of the uterine arteries, systemic MTX	Intentional interruption	Vaginal bleeding
Shah *et al*., 2009	34	IVF+ICSI	7w	CG aspiration (US guided), prophylactic placement of hypogastric arteries occlusion balloons before delivery	C-section (37w)	Asymptomatic / Bleeding after aspiration
Hoshino *et al.*, 2009	37	IVF	6w	CG extraction with forceps and curettage (US guided)	C-section (38w)	Vaginal bleeding
Kim *et al.*, 2009	30	Spontaneous	8w	CG aspiration (US guided), Foley catheter insertion	C-section (37w)	Asymptomatic / Bleeding during aspiration, cervical hematoma
Majumdar *et al*., 2009	36	IVF	7w 5d	Local injection of KCl (US guided)	C-section (31w)	Vaginal bleeding / IUGR, acute fetal distress
Nitke *et al.*, 2007	45	IVF		Uterine arteries MTX injection	Intentional interruption	Asymptomatic / Triplet gestation: 2 cervical gestational sacs
Suzuki *et al*., 2007	35	IVF	5w 3d	CG aspiration and local injection of 33% glucose solution	C-section (34w)	Asymptomatic / Bleeding, cervical hematoma
Prorocic & Vasiljevic, 2007	31	IVF	6w	CG aspiration and local injection of hypertonic solution of sodium chloride	Normal gestational course until 12w (time of report)	Vaginal bleeding / Triplet gestation: 2 IU embryos
Honda *et al*., 2005	40	IVF+ICSI	6w	Local injection of vasopressin, cervical curettage, local injection of MTX	Delivery (38w)	Vaginal bleeding
Feinberg & Confino, 2004	35	IVF	-	CG electro-cauterization (US guided) and extraction with forceps	Term vaginal delivery	Asymptomatic
Gyamfi *et al*., 2004	34	IVF	6w	Local injection of KCl and aspiration	C-section (31w)	Vaginal bleeding, back pain / Bleeding, hysterectomy, blood transfusion
Kumar *et al*., 2004	32	Spontaneous	7w	Local injection of KCl (US guided)	C-section (35w)	Bleeding / Impending eclampsia, bleeding, ligation of anterior division of both internal iliac arteries, blood transfusion
Jozwiak *et al.*, 2003	37	IVF+ICSI	7w 4d	CG electro-cauterization (US guided), cerclage (12w)	C-section (38w)	Asymptomatic
Oláh, 2003	34	IVF	12w	Local injection of KCl	C-section (36w)	Vaginal bleeding / Bleeding, hysterectomy, blood transfusion, DIC
Porpora *et al*., 2003	29	Spontaneous	6w	CG aspiration (US guided)	Abortion (1d after aspiration)	Asymptomatic / Bleeding
(Seow *et al*., 2002)	29	IVF	5w	CG extraction with forceps (US guided)	C-section (37w)	Vaginal bleeding, abdominal pain / Triplet gestation: 2 IU embryos
Mashiach *et al*., 2002	34	IVF	8w 3d	Cerclage (Shirodkar)	Vaginal delivery (39w)	-
Chen *et al.*, 2001	35	IVF	7w 4d	CG aspiration (US guided), local injection of KCl, cerclage (10w)	C-section (38w)	Vaginal bleeding / Bleeding
Al-azemi *et al*., 1999	32	IVF	6w	Local injection of KCl and MTX (US guided), aspiration	C-section (30w)	Vaginal bleeding / Bleeding after aspiration, premature amniorrhexis
Honey *et al.*, 1999	37	IVF	7w 4d	Uterine arteries embolization, local injection of KCl	Abortion	Vaginal bleeding / Bleeding, blood transfusion, chorioamnionitis, hysterectomy
Bratta *et al.*, 1996	30	-	7w	MTX IM (2x)	Intentional interruption	Asymptomatic / Bleeding
Peleg *et al.*, 1994	35	IVF	7w	MTX IM, uterine arteries MTX injection, uterine curettage	Intentional interruption	Vaginal bleeding / Bleeding, blood transfusion
Bayati *et al.*, 1989	38	IVF	11w 2d	Uterine aspiration and curettage, ligation of cervical artery	Intentional interruption	Vaginal bleeding

CG=cervical gestation; DIC=disseminated intravascular coagulation; ICSI=intracytoplasmic sperm injection; IUGR=intrauterine growth restriction; IM=intramuscular;  IU=intrauterine; IVF=*in vitro* fertilization; KCl=Chloride Potassium; MTX=methotrexate; US=ultrasound

Most of the case reports (n=31; 81.6%) resulted from IVF, in comparison to a minority (n=6; 15.8%), which resulted from spontaneous conception, and in one case the form of conception was not specified. Maternal age at diagnosis ranged from 25 to 45 years, with a mean of 34.6 years. The gestational age at the time of diagnosis ranged from 5 to 12 weeks, and except for Bayati *et al*. (1989), in which the diagnosis was made by direct visualization during the surgical approach, all cases were diagnosed via the US. As for clinical presentation, a small percentage was asymptomatic (n=12, 31.6%), and most of the cases presented vaginal bleeding at the time of diagnosis.

**Graph 1:**
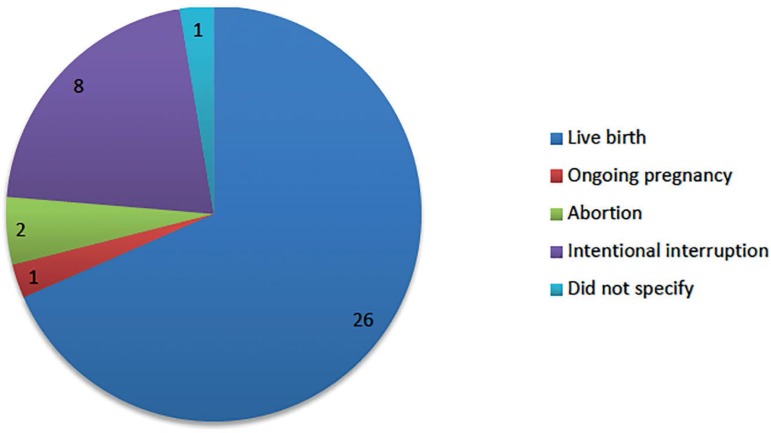
Heterotopic cervical pregnancies outcome (n=38)

Regarding the outcome (Graph 1), 29 cases aimed to maintain the IU pregnancy, opting for selective interruption of cervical pregnancy. Out of these, 26 resulted in live birth- nine of which were premature; two resulted in abortion and in one case, the pregnancy was still ongoing at the time of reporting. In eight cases, the two pregnancies were intentionally interrupted, and one of the reports did not specify the clinical outcome. The main complications were prematurity (n=9, 23.7%), severe bleeding with the need for blood transfusion (n=6, 15.8%), and emergency hysterectomy (n=4, 10.5%), in addition to cervical hematoma, placenta accreta, chorioamnionitis, intrauterine growth restriction (IUGR), and disseminated intravascular coagulation (DIC).

**Graph 2:**
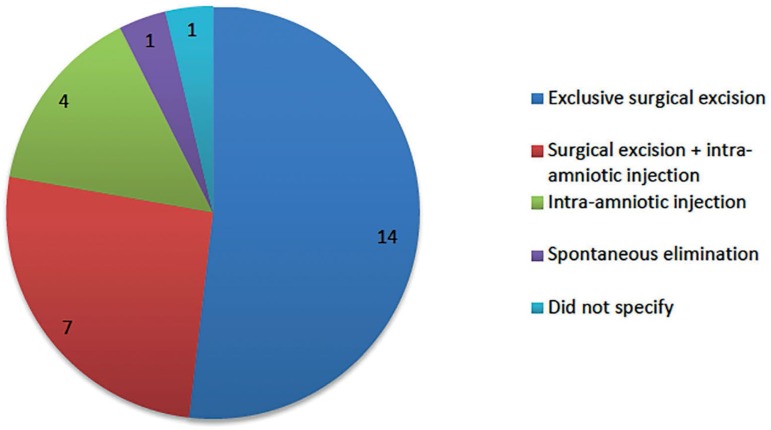
Treatment of heterotopic cervical pregnancies resulting in IU pregnancy maintenance (n=27)

Concerning the treatment, considering the cases which aimed to maintain the IU pregnancy (n=29) (Graph 2), the majority (n=23; 79.3%) preferred surgical excision of the cervical pregnancy. From most to least common, the surgical approaches included aspiration (n=12), extraction with forceps (n=6), cerclage (n=6), cervical curettage (n=5), Foley catheter insertion (n=4) and electrocauterization (n=3). In most cases, the procedures were guided by US, and in 10 cases, a combination of surgical treatments was performed. In eight cases, an association between surgical evacuation and intra-amniotic injection of KCl (n=4), MTX (n=2), hypertonic glucose (n=1) or sodium chloride (n=1) was used. In five cases, no surgical cervical evacuation was performed, with local injection of KCL (n=4) and MTX (n=1) - in one case there was an association with uterine artery embolization. It is noteworthy that the insertion of a Foley catheter, electrocautery and cervical injections of glucose and sodium chloride were never chosen as the single therapy, but rather combined with other methods. Of these 29 cases, in 27 the IU pregnancy was successfully maintained (Graph 2), and two cases resulted in abortion - one had been treated with an association of KCl injection and uterine artery embolization, and another with aspiration alone.

On the other hand, a minority of cases (n=8) opted for intentionally interrupting both cervical and IU pregnancies. Those were treated with systemic MTX (n=6), uterine artery embolization (n=3), uterine curettage (n=3), aspiration (n=2) and Foley catheter insertion (n=1). In most cases, a combined therapy was performed, and there was one report of exclusive treatment with systemic MTX.

In the current report, we present the first Brazilian report of cervical heterotopic pregnancy after IVF. The patient presented with secondary infertility, with history of repeated abortion, who underwent 6 cycles of IVF. Only after few transferred blastocysts, with a donor oocyte, the pregnancy was achieved. The patient was asymptomatic when we made the diagnosis of cervical heterotopic pregnancy, at the tenth week of gestational age. We chose to selectively reduce the cervical embryo with KCl injection guided by US, aiming to maintain the IU gestation. However, after patient sedation and introduction of the vaginal speculum, there was spontaneous elimination of the cervical embryo, with preservation of IU gestation.

Finally, according to the current literature, most cases of cervical heterotopic pregnancy have a favorable outcome, resulting in live birth and maintenance of maternal fertility after selective reduction of the cervical embryo. However, it is possible that this analysis is overly optimistic, since experiences with an unfavorable outcome are more unlikely to be reported.

## CONCLUSION

In conclusion, according to recent literature, it may be suggested that cervical heterotopic pregnancy should be treated through US-guided surgical excision associated or not with KCl intra-amniotic injection, once most of the favorable outcomes have been achieved in this manner. However, it is worth mentioning that the number of literature reports is still insufficient to safely establish the best treatment for this condition. Thus, the treatment should be individually chosen considering the patient's will to maintain the IU pregnancy and the personal experience of the doctor in charge.

## References

[r1] Al-Azemi M, Ledger WL, Lockwood GM, Barlow DH (1999). Successful transvaginal ultrasound-guided ablation of a cervical
pregnancy in a patient with simultaneous intrauterine pregnancy after in
vitro fertilization and embryo transfer. Hum Fertil (Camb).

[r2] Bayati J, Garcia JE, Dorsey JH, Padilla SL (1989). Combined intrauterine and cervical pregnancy from in vitro
fertilization and embryo transfer. Fertil Steril.

[r3] Bratta FG, Ceci O, Loizzi P (1996). Combined intra-uterine and cervical pregnancy treated
successfully with methotrexate. Int J Gynaecol Obstet.

[r4] Chen D, Kligman I, Rosenwaks Z (2001). Heterotopic cervical pregnancy successfully treated with
transvaginal ultrasound-guided aspiration and cervical-stay
sutures. Fertil Steril.

[r5] Deka D, Bahadur A, Singh A, Malhotra N (2012). Successful management of heterotopic pregnancy after fetal
reduction using potassium chloride and methotrexate. J Hum Reprod Sci.

[r6] Dibble EH, Lourenco AP (2016). Imaging Unusual Pregnancy Implantations: Rare Ectopic Pregnancies
and More. AJR Am J Roentgenol.

[r7] Elena HE, Elena AF, Miola A, Glujovsky D, Sueldo CE (2016). Successful treatment of a cervical heterotopic pregnancy
following an n vitro fertilization procedure. Medicina (Buenos Aires).

[r8] Faschingbauer F, Mueller A, Voigt F, Beckmann MW, Goecke TW (2011). Treatment of heterotopic cervical pregnancies. Fertil Steril.

[r9] Feinberg E, Confino E (2004). Electrodessication of a cervical heterotopic
pregnancy. Fertil Steril.

[r10] Gyamfi C, Cohen S, Stone JL (2004). Maternal complication of cervical heterotopic pregnancy after
successful potassium chloride fetal reduction. Fertil Steril.

[r11] Hafner T, Ivkosic IE, Serman A, Bauman R, Ujevic B, Vujisic S, Hafner D, Miskovic B (2010). Modification of conservative treatment of heterotopic cervical
pregnancy by Foley catheter balloon fixation with cerclage sutures at the
level of the external cervical os: a case report. J Med Case Rep.

[r12] Honda R, Matsuura K, Okamura H (2005). Heterotopic cervical pregnancy with preservation of the
intrauterine gestation. Reprod Med Biol.

[r13] Honey L, Leader A, Claman P (1999). Uterine artery embolization--a successful treatment to control
bleeding cervical pregnancy with a simultaneous intrauterine
gestation. Hum Reprod.

[r14] Hoshino T, Kita M, Imai Y, Kokeguchi S, Shiotani M (2009). Successful pregnancy outcome in a case of heterotopic
intrauterine and cervical pregnancy and a literature review. J Obstet Gynaecol Res.

[r15] Jozwiak EA, Ulug U, Akman MA, Bahceci M (2003). Successful resection of a heterotopic cervical pregnancy
resulting from intracytoplasmic sperm injection. Fertil Steril.

[r16] Kim MG, Shim JY, Won HS, Lee PR, Kim A (2009). Conservative management of spontaneous heterotopic cervical
pregnancy using an aspiration cannula and pediatric Foley
catheter. Ultrasound Obstet Gynecol.

[r17] Kim JW, Park HM, Lee WS, Yoon TK (2012). What is the best treatment of heterotopic cervical pregnancies
for a successful pregnancy outcome?. Clin Exp Reprod Med.

[r18] Kumar S, Vimala N, Dadhwal V, Mittal S (2004). Heterotopic cervical and intrauterine pregnancy in a spontaneous
cycle. Eur J Obstet Gynecol Reprod Biol.

[r19] Lin CK, Wen KC, Sung PL, Lin SC, Lai CR, Chao KC, Yen MS, Chen CC, Li HY, Too LL (2013). Heterotopic triplet pregnancy with an intrauterine, a tubal, and
a cervical gestation following in vitro fertilization and embryo
transfer. Taiwan J Obstet Gynecol.

[r20] Majumdar A, Gupta SM, Chawla D (2009). Successful management of post-in-vitro fertilization cervical
heterotropic pregnancy. J Hum Reprod Sci.

[r21] Mashiach S, Admon D, Oelsner G, Paz B, Achiron R, Zalel Y (2002). Cervical Shirodkar cerclage may be the treatment modality of
choice for cervical pregnancy. Hum Reprod.

[r22] Molinaro TA, Barnhart KT (2018). Abdominal pregnancy, cesarean scar pregnancy, and heterotopic
Pregnancy. UpToDate®.

[r23] Moragianni VA, Hamar BD, McArdle C, Ryley DA (2012). Management of a cervical heterotopic pregnancy presenting with
first-trimester bleeding: case report and review of the
literature. Fertil Steril.

[r24] Nitke S, Horowitz E, Farhi J, Krissi H, Shalev J (2007). Combined intrauterine and twin cervical pregnancy managed by a
new conservative modality. Fertil Steril.

[r25] Oláh KS (2003). Massive obstetric haemorrhage resulting from a conservatively
managed cervical pregnancy at delivery of its twin. BJOG.

[r26] Peleg D, Bar-Hava I, Neuman-Levin M, Ashkenazi J, Ben-Rafael Z (1994). Early diagnosis and successful nonsurgical treatment of viable
combined intrauterine and cervical pregnancy. Fertil Steril.

[r27] Pinto BB, Torres TP, Narváez MB, Rojas XB, Burgos IA, Constante PE (2016). Heterotopic cervical pregnancy management after a high-complexity
assisted reproduction procedure. JBRA Assist Reprod.

[r28] Porpora MG, D'Elia C, Bellavia M, Pultrone DC, Cosmi EV (2003). Heterotopic cervical pregnancy: a case report. Acta Obstet Gynecol Scand.

[r29] Prorocic M, Vasiljevic M (2007). Treatment of heterotopic cervical pregnancy after in vitro
fertilization-embryo transfer by using transvaginal ultrasound-guided
aspiration and instillation of hypertonic solution of sodium
chloride. Fertil Steril.

[r30] Punhani R, Shankar K, Varma TR (2016). A rare and interesting case of heterotopic cervical pregnancy
after intracytoplasmic sperm injection and embryo transfer. J Hum Reprod Sci.

[r31] Reece EA, Petrie RH, Sirmans MF, Finster M, Todd WD (1983). Combined intrauterine and extrauterine gestations: a
review. Am J Obstet Gynecol.

[r32] Saito K, Fukami M, Miyado M, Ono I, Sumori K (2018). Case of heterotopic cervical pregnancy and total placenta accreta
after artificial cycle frozen-thawed embryo transfer. Reprod Med Biol.

[r33] Sánchez-Ferrer ML, Machado-Linde F, Pertegal-Ruiz M, García-Sánchez F, Pérez-Carrión A, Capel-Aleman A, Parilla-Paricio JJ, Abad-Martínez L (2011). Fertility preservation in heterotopic cervical pregnancy: what is
the best procedure?. Fetal Diagn Ther.

[r34] Seow KM, Hwang JL, Tsai YL, Lin YH, Hsieh BC, Huang SC (2002). Transvaginal colour Doppler diagnosis and assessment of a
heterotopic cervical pregnancy terminated by forceps evacuation following in
vitro fertilisation and embryo transfer. BJOG.

[r35] Shah AA, Grotegut CA, Likes CE 3rd, Miller MJ, Walmer DK (2009). Heterotopic cervical pregnancy treated with transvaginal
ultrasound-guided aspiration resulting in cervical site varices within the
myometrium. Fertil Steril.

[r36] Sijanovic S, Vidosavljevic D, Sijanovic I (2011). Methotrexate in local treatment of cervical heterotopic pregnancy
with successful perinatal outcome: case report. J Obstet Gynaecol Res.

[r37] Subedi J, Xue M, Sun X, Xu D, Deng X, Yu K, Yang X (2016). Hysteroscopic management of a heterotopic pregnancy following
uterine artery embolization: a case report. J Med Case Rep.

[r38] Suzuki M, Itakura A, Fukui R, Kikkawa F (2007). Successful treatment of a heterotopic cervical pregnancy and twin
gestation by sonographically guided instillation of hyperosmolar
glucose. Acta Obstet Gynecol Scand.

[r39] Tsakos E, Tsagias N, Dafopoulos K (2015). Suggested Method for the Management of Heterotopic Cervical
Pregnancy Leading to Term Delivery of the Intrauterine Pregnancy: Case
Report and Literature Review. J Minim Invasive Gynecol.

[r40] Uysal F, Uysal A (2013). Spontaneous heterotopic cervical pregnancy and successful
pregnancy outcome. J Ultrasound Med.

